# Effect of graphene oxide dosage on the thermal and rheological behavior of asphalt for tropical road conditions

**DOI:** 10.3389/fchem.2025.1691517

**Published:** 2025-10-17

**Authors:** Erick Mendoza, Talia Tene, Jorge Albuja-Sánchez, Guisella Cuenca, John Ramón, David Anzules, Cristian Vacacela Gomez, Yolenny Cruz Salazar, Lorenzo S. Caputi, Salvatore Straface

**Affiliations:** 1 Maestría en Física, Facultad de Posgrado, Universidad Técnica de Manabí, Portoviejo, Manabí, Ecuador; 2 Departament of Chemistry, Universidad Técnica Particular de Loja, Loja, Ecuador; 3 Multidisciplinary Engineering Research Hub (MER Hub), Faculty of Habitat, Infrastructure, and Creativity, Pontificia Universidad Católica del Ecuador (PUCE), Quito, Ecuador; 4 International Faculty of Innovation PUCE-Icam, Pontificia Universidad Católica del Ecuador (PUCE), Quito, Ecuador; 5 Ingeniería Química, Facultad de Ciencias Exactas y Naturales, Universidad Técnica Particular de Loja, Loja, Ecuador; 6 Departament of Physics, Facultad de Ciencias Básicas, Universidad Técnica de Manabí, Portoviejo, Manabí, Ecuador; 7 Department of Environmental Engineering, DIAm, University of Calabria, Rende, Calabria, Italy; 8 Universidad Ecotec, Samborondón, Ecuador; 9 Surface Nanoscience Group, Department of Physics, University of Calabria, Rende, Italy; 10 UNICARIBE Research Center, University of Calabria, Rende, Italy

**Keywords:** asphalt, graphene oxide, viscosity, hardness, resistance

## Abstract

This study investigates graphene oxide (GO) as a low-percent modifier for AC-30 asphalt used in tropical conditions. GO was added at 0.2, 0.4, and 0.6 wt% and tested through standard binder tests and RTFO short-term aging. Unaged binders with GO showed increased viscosity and hardness—viscosity rose by up to approximately 26%, penetration decreased by about 8%–13%, and the softening point rose slightly—indicating a stiffer initial matrix. During mixing at 135 °C–165 °C, both rotational and kinematic viscosities increased, with the highest gains near 0.4 wt%, suggesting a non-linear response to dosage. Under RTFO aging, GO-modified binders exhibited a higher viscosity aging index than the unmodified AC-30, indicating that GO enhances initial stiffness and does not compromise short-term oxidative hardening. Flash and fire points did not show systematic reductions at these dosages. Overall, sub-percent GO improves high-temperature rheology relevant for hot-climate pavements, but there is a trade-off between better early rutting resistance and potential stiffening with aging. Future research will include PAV/DSR testing and analysis of dispersion better to understand long-term behavior and processing at the plant level.

## 1 Introduction

Asphalt is a viscoelastic material commonly used in road construction because of its mechanical and adhesive properties (Speight, 2013). However, despite the positive qualities of virgin asphalt, early pavement deterioration remains a consistent problem, caused by various environmental and mechanical stressors (Menozzi et al., 2015; Omar et al., 2020). To improve the quality and lifespan of asphalt binders, the addition of nanomaterials as modifying agents has attracted significant interest in recent years (Cai et al., 2023). The use of nanomaterials covers multiple scientific fields (Jeevanandam et al., 2019; Jimenez Rodríguez et al., 2019; Tene et al., 2023), and their application in asphalt technology has yielded promising results, especially those based on graphene derivatives (Induchoodan et al., 2021; Wu and Tahri, 2021).

Among these, graphene oxide (GO) has become a notable nanomaterial for asphalt modification (Adnan et al., 2020). GO is a two-dimensional nanostructure composed of atomically thin layers functionalized with oxygen-containing groups (Gholampour et al., 2017; Guo et al., 2022; Kashif et al., 2021), which endows it with excellent mechanical, electronic, optical, and chemical properties (Zhao et al., 2015). Its versatility has led to wide applications in biology, environmental science, electronics, and medicine (Chung et al., 2013). Though its synthesis was once complex and costly, advancements in technology have made GO more accessible for commercial use (Hernandez et al., 2018; Tene et al., 2020). These unique physicochemical properties have driven extensive research into its role as an asphalt modifier (Xu et al., 2023).

Research has shown that when GO is mixed with asphalt, no chemical reaction takes place between the two components (Li et al., 2019; Yu et al., 2019). Instead, GO’s structure allows for a type of intercalation with specific asphalt molecules, effectively blending the materials (Zhu et al., 2020). The oxygen-containing functional groups in GO can form hydrogen bonds with asphalt components, creating a structurally advantageous connection (Sengottuvelu et al., 2024; Si et al., 2023). Additionally, π-π stacking interactions are thought to occur between GO’s aromatic regions and the aromatic compounds in asphalt (Zeng et al., 2020). This molecular-level interaction improves the colloidal stability of the asphalt matrix by increasing the density of its internal network (Li et al., 2022; Wei et al., 2024).

These microstructural changes directly affect the physical, mechanical, and rheological properties of the asphalt binder. GO increases both the surface free energy and surface area of the asphalt, promoting stronger adhesion and better interactions with aggregate materials (Zhang et al., 2017). Furthermore, it enhances viscoelasticity and rigidity, which leads to improved performance under mechanical loads and thermal stress (Li et al., 2023). GO has also been shown to delay the oxidative aging of asphalt, thereby extending pavement lifespan (Wang et al., 2019; Wu et al., 2017). Meanwhile, its hydrophobic character enhances moisture resistance—an advantage for infrastructure in humid or rainy climates (He et al., 2022; Zhu et al., 2019). Additionally, incorporating GO improves resistance to deformation, cracking, and fatigue, all of which contribute to longer-lasting road surfaces (Adnan et al., 2023; An et al., 2023; Liu et al., 2018). From a thermodynamic perspective, GO provides better stability at high temperatures, making it suitable for hot climates (Qian et al., 2022; Wang et al., 2020). However, a notable drawback is the observed reduction in ductility at low temperatures as the GO content increases, a limitation reported in several studies (Jiang et al., 2022; Zhang et al., 2022).

Despite extensive research, the optimal dosage of GO for asphalt modification remains debated. For example, W. Zeng et al. reported that a 1% GO content produced the best results among 1% and 3% dosages (Zeng et al., 2017), while S. Wu et al. found better outcomes with a 3% addition using the same concentrations (Wu et al., 2017). Zhu et al. showed that as little as 0.2% GO could significantly enhance surface free energy and decrease oxidative aging (Zhu et al., 2020). Singh et al. observed that 1% GO was most effective against rutting, whereas 2% provided greater resistance to fatigue (Singh et al., 2020). Adnan et al. evaluated GO contents from 0% to 2.5%, concluding that 2% offered the best performance improvements (Adnan et al., 2021). Similarly, F. Wu et al. found 1% to be the most effective for high-temperature performance among tested values of 0%–2% (Wu et al., 2022). A more recent study by Sengottuvelu et al. indicated that 2% GO caused the most significant changes in asphalt properties compared to lower concentrations (Sengottuvelu et al., 2024). This variation across studies highlights that the ideal GO content depends on factors such as dispersion method, the chemical makeup of the base asphalt, and specific testing conditions.

In Ecuador, the main source of asphalt for road construction is the Esmeraldas Refinery. However, the bitumen it provides is known for its comparatively low durability and early aging, despite regulatory efforts to ensure quality (Vil et al., 2021). Several studies have aimed to enhance the performance of Ecuadorian asphalt (Chávez- Romero et al., 2019; Navarrete Schettini, 2019; Vil et al., 2021), yet—to our knowledge—no local research has examined GO as a modifier for AC-30 under tropical service conditions using sub-percent dosages and short-term aging (RTFO) benchmarks. The current study presents the first Ecuadorian assessment of GO-modified asphalt, determines the dosage–response related to local plant temperatures, and outlines application pathways for hot-climate pavements, thereby filling a regional knowledge gap and establishing a practical baseline for future field validation.

## 2 Materials and methods

### 2.1 Materials

The asphalt binder used in this study was obtained from the Uruzca quarry in Portoviejo, Manabí, Ecuador (1.057386° S, 80.565821° W), which is supplied with bitumen from the Esmeraldas Refinery. Graphene oxide (GO) was synthesized in-house at Universidad Técnica Particular de Loja (UTPL) following our previously reported eco-friendly protocol (Tene et al., 2020). Details of spectroscopic and morphological characterization of GO (e.g., Raman, UV–Vis, SEM, TEM, and others) are provided in Ref. (Tene et al., 2020).

We point out again that the base binder (AC-30) was procured from Refinería Esmeraldas; however, SARA fractionation (saturates, aromatics, resins, and asphaltenes) was not performed, as the present study focuses on the thermal and rheological responses to graphene oxide dosage under tropical service conditions. Source variability was addressed operationally by using a consistent supplier and procurement channel during the experimental campaign. Refinería Esmeraldas publishes routine quality reports (MTOP Ministerio de Transporte y Obras Públicas, 2013) indicating narrow inter-batch ranges for key binder properties. Additionally, standardized handling and storage procedures were implemented to minimize material drift before modification. This combination of controls supports attribution of the observed trends primarily to GO dosage rather than to uncontrolled binder variability. As a perspective, future work will incorporate SARA fractionation and multi-batch comparisons to link colloidal composition with the measured rheological responses.

### 2.2 Preparation of GO

**FIGURE 1:**
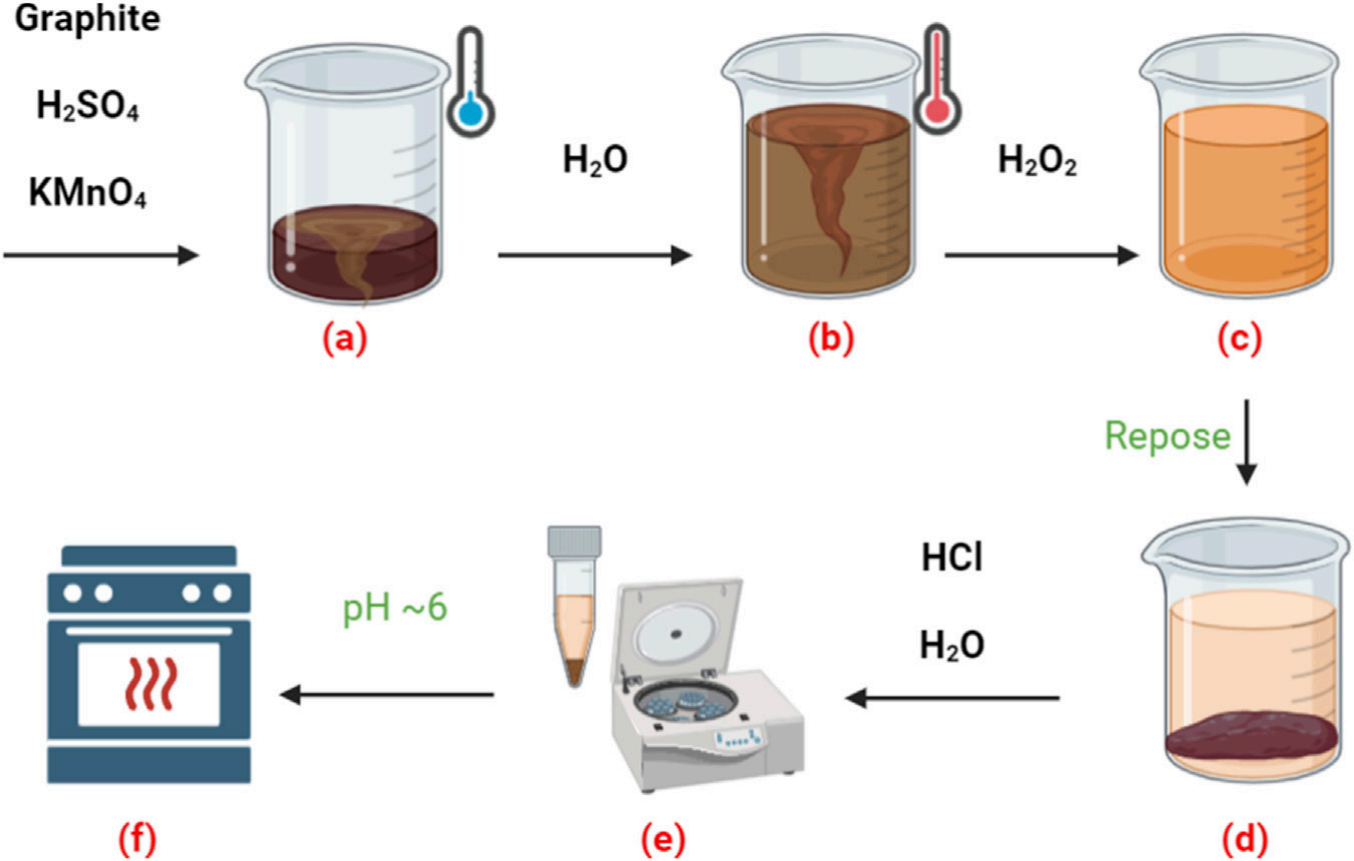
Illustration of GO formation process: **(a)** Oxidation, **(b)** water interaction, **(c)** peroxide addition, **(d)** precipitation, **(e)** purification and centrifugation, and **(f)** drying of the GO sample.

The synthesis of GO in this study followed a several-step process based on a modified Hummer’s method (see Figure 1).

- The process began by gradually adding 70 mL of concentrated H_2_SO_4_ to 3 g of graphite powder while continuously stirring, maintaining a temperature below 20 °C to prevent unwanted side reactions. Stirring continued for 10 min to ensure thorough mixing. Next, 9 g of KMnO_4_ was slowly added while stirring for another 30 min (Step a).
- In the second step, the mixture was gently heated to 50 °C and stirred for another 30 min. Then, 150 mL of distilled water was added dropwise, carefully controlling the addition rate to maintain a temperature below 90 °C. Stirring continued for 20 min to ensure even dispersion. Afterward, 500 mL of distilled water was added to dilute the mixture (Step b) further.
- To prevent oxidation, 15 mL of H_2_O_2_ was added immediately. The reaction was deemed complete once the mixture turned a uniform amber color, at which point stirring was stopped (Step c). The resulting suspension was left undisturbed for 24 h to allow graphite oxide to precipitate (Step d).
- The precipitate was then subjected to a series of purification steps, including repeated washing and centrifugation cycles (8 min each at 3000 rpm). The first wash used a 1:10 HCl solution, followed by several rinses with distilled water until the pH of the suspension reached about 6 (Step e). Finally, the purified material was dried at 80 °C for 24 h to produce solid GO suitable for asphalt modification (Step f).

Notably, GO was synthesized using our eco-friendly, aqueous method that avoids the strong chemical oxidation typical of Hummers-type procedures (Tene et al., 2020). The product was washed with deionized water to reach a near-neutral pH (≈6) before drying. This process reduces residual acidic species. Then, no corrosion issues are expected to be caused by acid carryover at the sub-percent GO dosages used.

### 2.3 Preparation of asphalt samples

**FIGURE 2:**
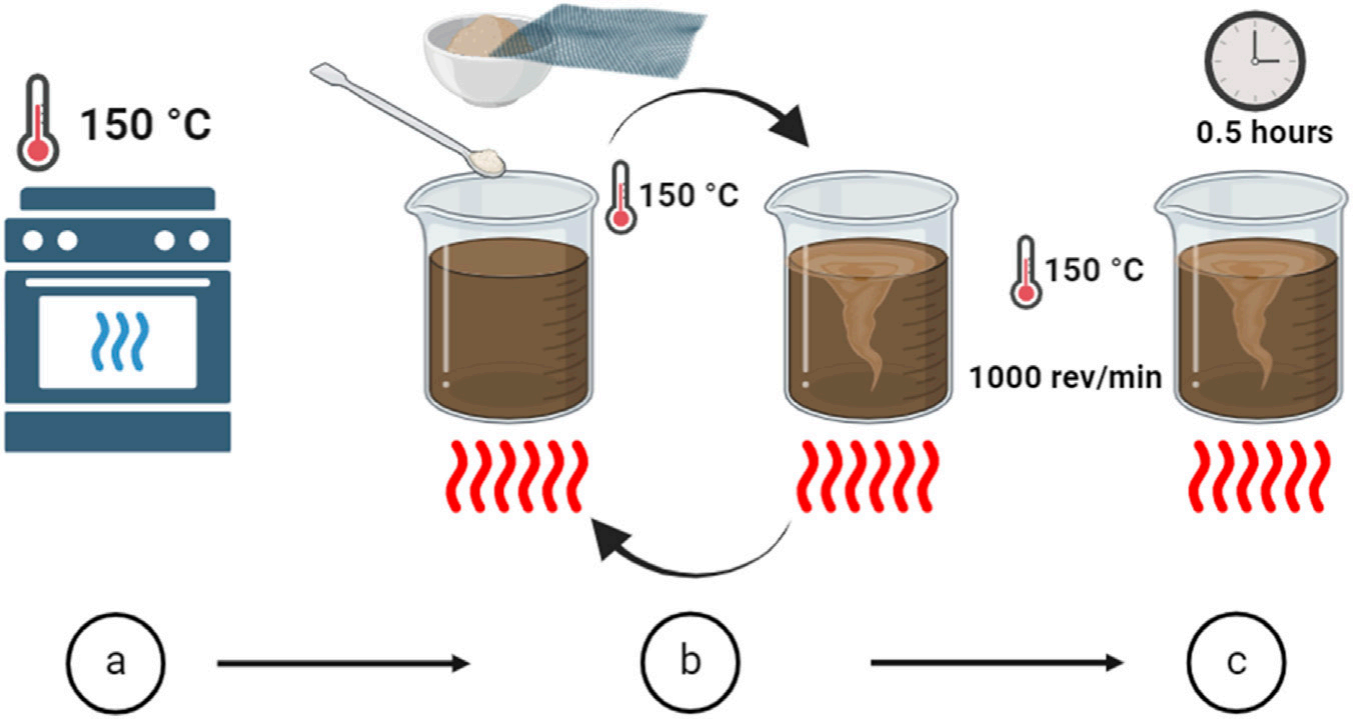
Illustration of the formation process of GO-modified samples, see detail in the main text for each step from **(a)** to **(c)**.

The process for preparing GO-modified asphalt involved three distinct steps Figure 2. First, 600 g of base asphalt (BA) was slowly heated to 150 °C until it was fully melted and uniform (Step a). Then, the predetermined amount of GO was gradually added to the molten asphalt. After each addition, the mixture was stirred continuously at 1000 revolutions per minute to ensure even distribution. This process was repeated until all the GO was incorporated (Step b). Finally, the asphalt-GO mixture was kept stirring at 150 °C for another 30 min to achieve thorough homogenization of the nanomaterial within the asphalt matrix (Step c).

In this study, four asphalt samples were prepared for comparative evaluation. One unmodified BA and three samples modified with increasing amounts of GO.

GO dosages of 0.2, 0.4, and 0.6 wt% were chosen to cover a sub-percent, realistic plant range that has been shown to provide measurable benefits without causing unacceptable viscosity or dispersion issues. A consistent 0.2% increase was used to identify potential non-linear trends within the early screening scope. The exact quantities of material used for each formulation are listed in Table 1.

**TABLE 1:** Quantitative Formulation of Asphalt-GO samples.

Factor	BA	0.2GO	0.4GO	0.6GO
Mass of Asphalt (g)	600.00	600.00	600.00	600.00
Mass of GO (g)	0.00	1.20	2.40	3.60
Total mass (g)	600.00	601.20	602.40	603.60

To ensure consistency and clarity throughout the manuscript, the modified samples are labeled as follows: 0.2GO, 0.4GO, and 0.6GO, corresponding to 0.2%, 0.4%, and 0.6% GO by weight, respectively.

For tests involving aged asphalt, the same naming structure is maintained by prefixing each sample with an “A.”, resulting in A.BA, A.0.2GO, A.0.4GO, and A.0.6GO. The aging process of the samples was conducted following ASTM D2872.

### 2.4 Microstructural characterization

To analyze the morphology and elemental composition of the synthesized GO and its interaction with the asphalt matrix, one GO sample and one GO-modified asphalt sample were selected. Two randomly chosen samples were examined using scanning electron microscopy (SEM) and energy-dispersive X-ray spectroscopy (EDS).

### 2.5 Test methods

The asphalt samples were tested following recognized ASTM (American Society for Testing and Materials) standards. The tests, their corresponding ASTM designations, and the specific test conditions are summarized in Table 2.

**TABLE 2:** Tests carried out on asphalt samples.

No.	Test description	ASTM standard	Test conditions
1	Absolute Viscosity at 60 °C^a^	ASTM D2171	Standard conditions
2	Softening Point^b^	ASTM D36	Using distilled water
3	Penetration and Penetration Index	ASTM D5	Standard loading and timing
4	Ductility^c^	ASTM D113	25 °C, elongation at 5 cm/min
5	Specific Gravity and Density	ASTM D70	25 °C/25 °C (sample/water)
6	Brookfield Rotational Viscosity at 135 °C	ASTM D4402	Standard conditions
7	Flash and Fire Point	ASTM D92	Standard conditions
8	Mass Change from RTFO Aging	ASTM D2872	Standard conditions

^a^ Pre and Post-RTFO.

^b^ Ring-and-Ball Method.

^c^ Post-RTFO.

### 2.6 Statistical analysis

A one-way analysis of variance (ANOVA) was used to determine if there were statistically significant differences among the GO-modified asphalt samples and unmodified BA. To further analyze differences between the modified samples and the control BA, a *post hoc* Dunnett test was performed. Statistical calculations were conducted using IBM SPSS Statistics.

## 3 Results

### 3.1 Characterization

Although the Esmeraldas Refinery classifies its bitumen as AC-20, laboratory tests in this study showed discrepancies with this classification. Specifically, according to the standards set by the Ministerio de Transporte y Obras Públicas (MTOP), the BA did not meet the criteria for AC-20. Instead, its properties were more consistent with those of the AC-30 category (MTOP Ministerio de Transporte y Obras Públicas, 2013) (see Table 3).

**TABLE 3:** Specifications of the MTOP and characterization of the BA sample. Modified based on Ref (MTOP Ministerio de Transporte y Obras Públicas, 2013).

Test	Specification AC-20	Specification AC-30	Characteristics BA	Unit
Absolute Viscosity at 60 °C	200 ± 40	300 ± 60	248.35 ± 9.577	Pa.s
Brookfield rotational viscosity at 135 °C	≥300	≥350	410.4 ± 0.66	mm^2^/s
Penetration at 25 °C, 100 g, 5s	≥60	≥50	75.5 ± 1.43	0.1 mm
Flash point	≥230	≥230	267 ± 0.5	°C
Rolling thin-film oven test				
Ductility at 25 °C, 5 cm/min	≥50	≥40	44.5 ± 0.25	cm

**FIGURE 3:**
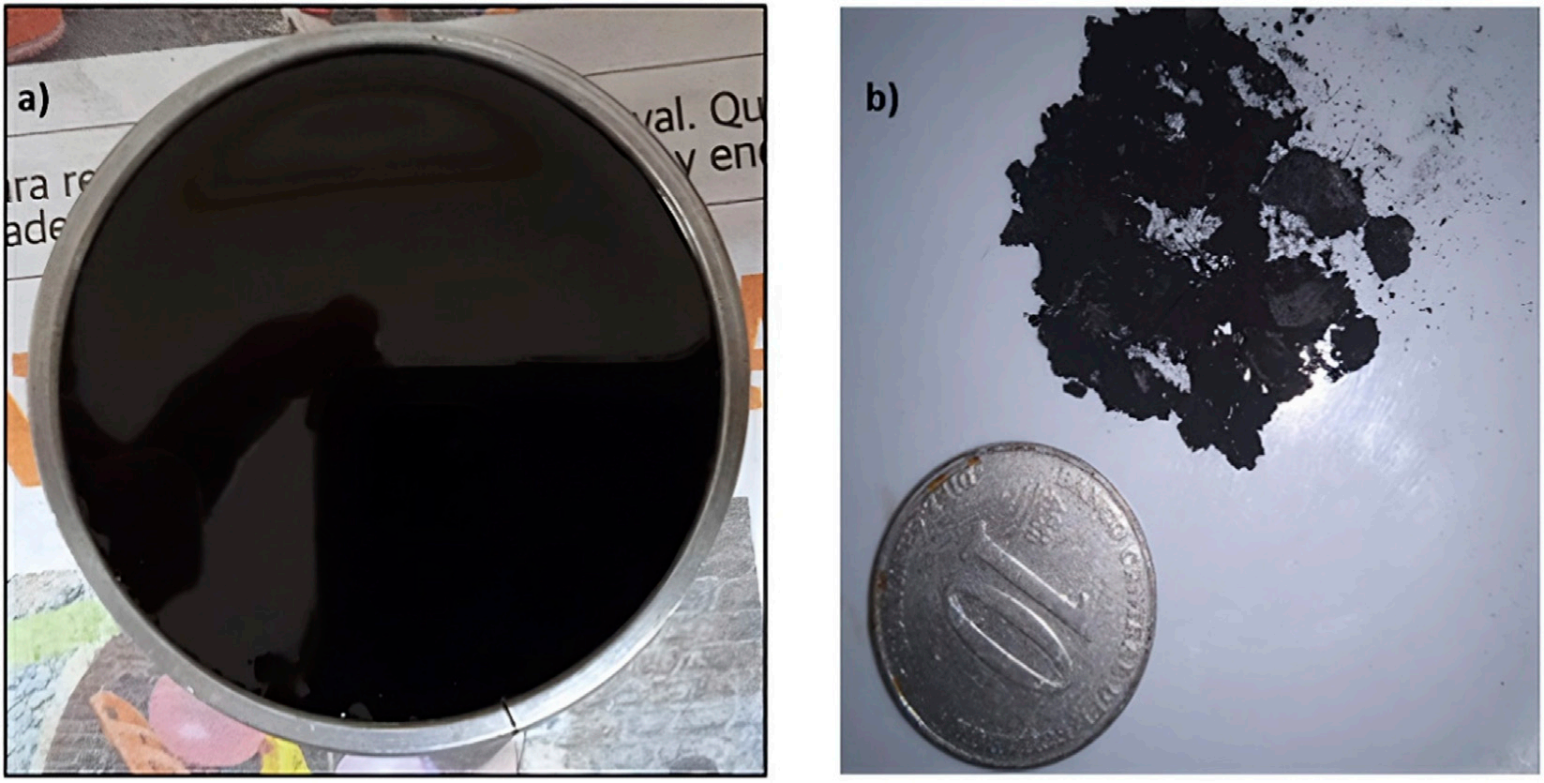
Appearance of **(a)** BA and **(b)** GO compared to a ten-cent coin.

Regarding physical characteristics, BA appeared as a black, semi-solid, viscous substance with a smooth surface (Figure 3a). Graphene oxide (GO) showed a heterogeneous appearance, mainly composed of black powder mixed with fine, thin gray flakes (Figure 3b). The synthesized GO used in this research had a pH of about 6, indicating a mildly acidic surface chemistry suitable for interaction with bitumen.

### 3.2 SEM and EDS results

**FIGURE 4:**
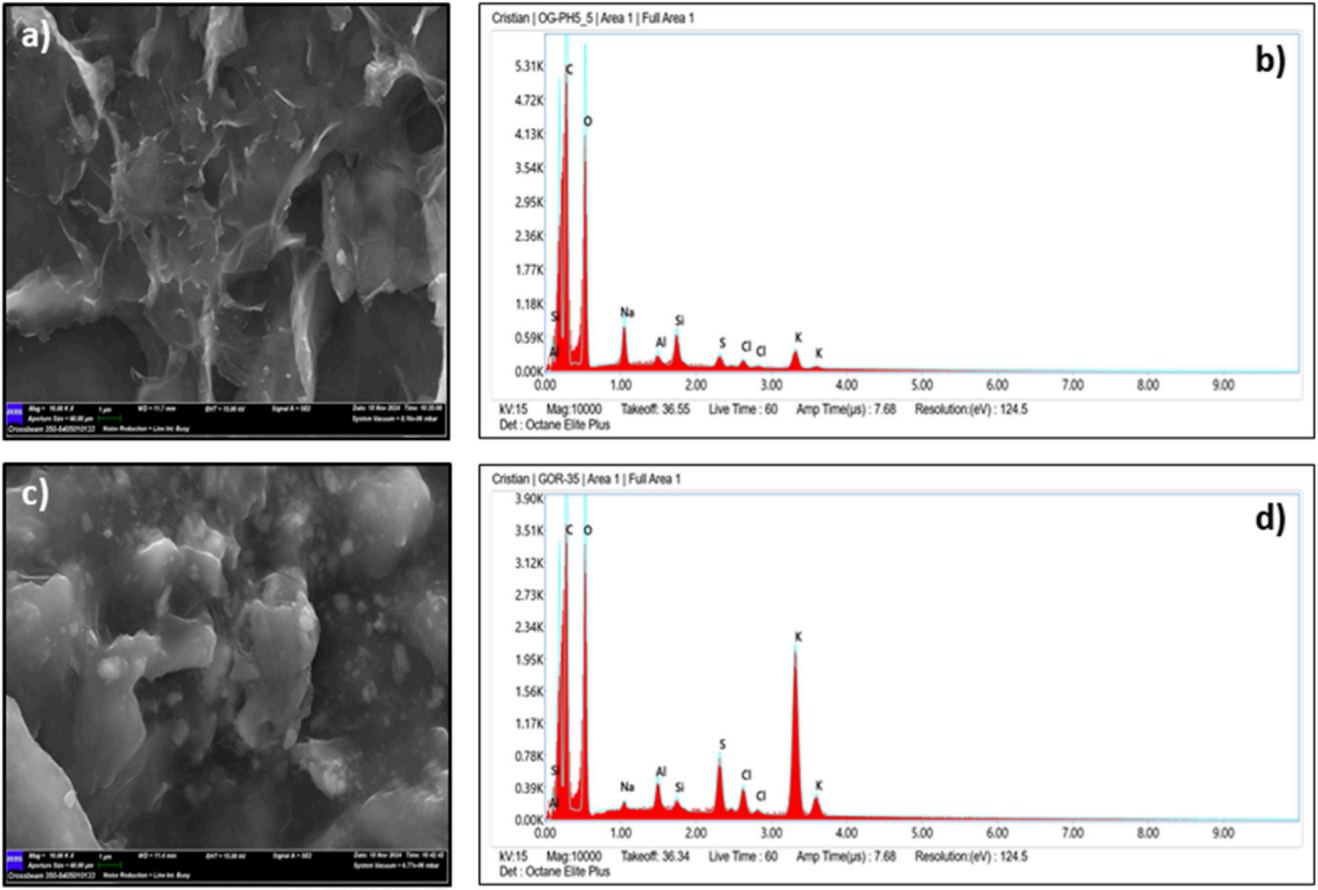
SEM and EDS Characterization of **(a,b)** GO and **(c,d)** GO-modified asphalt.

The SEM micrograph of GO (Figure 4a) showed a characteristic morphology of folded, wrinkled, and overlapping layers that are randomly arranged—features commonly linked to exfoliated GO sheets. EDS analysis (Figure 4b) confirmed that this sample mainly consists of carbon (61.9%) and oxygen (32.3%), with trace elements including sodium (1.9%), aluminum (0.4%), silicon (1.0%), sulfur (0.5%), chlorine (0.4%), and potassium (1.5%).

The second sample, the GO-modified asphalt sample, showed a somewhat different surface structure under SEM (Figure 4c), characterized by rough folds and fragmented grains. Its EDS profile (Figure 4d) also highlighted high concentrations of carbon (51.6%) and oxygen (32.9%), but with a significantly higher potassium content (10.9%). Other detected elements included sodium (0.3%), aluminum (0.8%), silicon (0.3%), sulfur (2.0%), and chlorine (1.2%).

In general, both samples confirmed the successful synthesis of GO with a mainly folded sheet-like structure. However, minor imperfections and heterogeneities, such as those seen in the GO-modified asphalt sample, are probably due to residual effects from the oxidizing agent KMnO_4_ used during synthesis. The differences in EDS results between the two samples indicate a localized distribution of oxygen-containing functional groups, which could affect GO’s interaction with the asphalt matrix.

### 3.3 Test results

#### 3.3.1 Test 1: absolute viscosity at 60 °C

**FIGURE 5:**
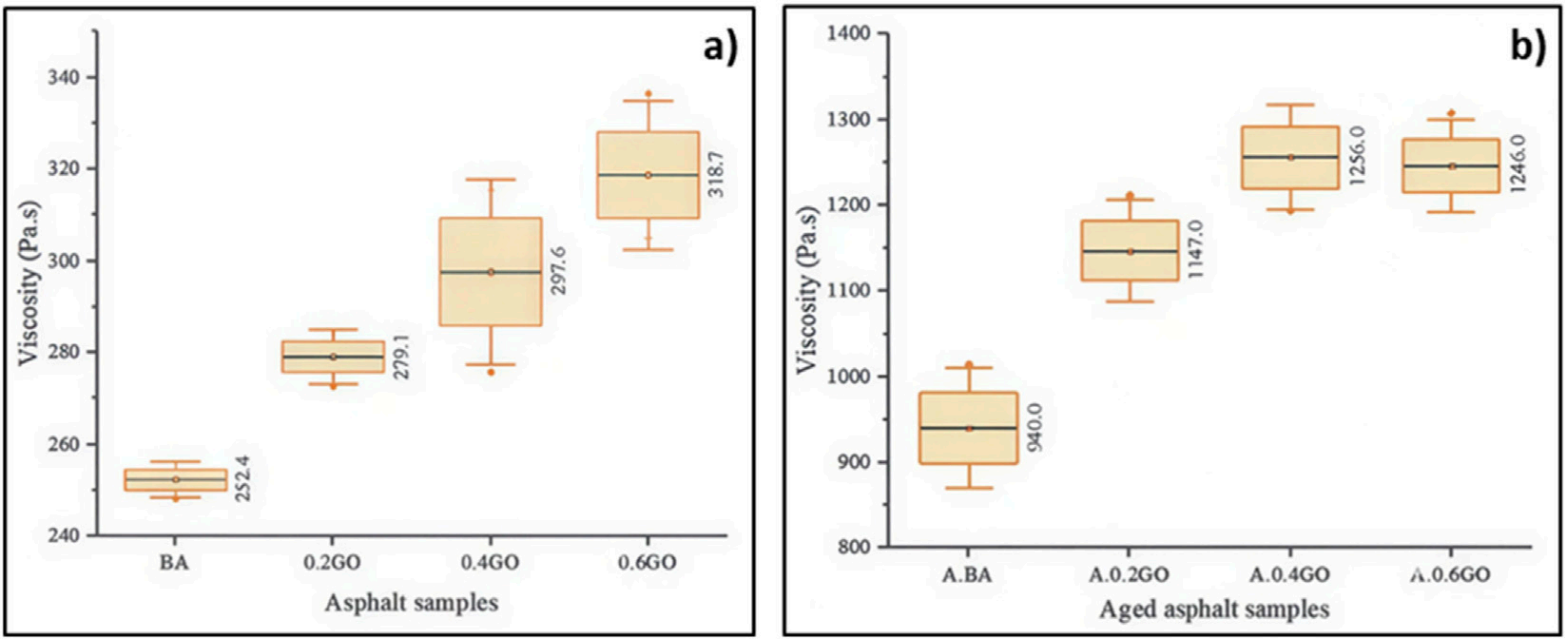
Viscosity test results at 60 °C for **(a)** unaged and **(b)** aged asphalt samples.

Figure 5a shows the viscosity measurements at 60 °C for the unaged asphalt samples. A clear upward trend in viscosity is observed as the proportion of GO increases in the mixture. Compared to the average viscosity of the BA, adding GO at concentrations of 0.2%, 0.4%, and 0.6% caused viscosity increases of 10.58%, 17.90%, and 26.27%, respectively. The highest viscosity was recorded for the 0.6GO sample. It is noteworthy that the 0.4GO and 0.6GO samples displayed greater data variability, likely due to the sensitivity of the measurement technique at higher viscosities. In contrast, the BA and 0.2GO samples showed more consistent results.

Figure 5b shows the viscosity results at 60 °C for aged asphalt samples. Like the unaged samples, viscosity increased with higher GO content. However, a slight decrease in viscosity was observed when moving from A.0.4GO to A.0.6GO, a phenomenon also noted in previous studies. Compared to the aged base asphalt (A.BA), adding GO at 0.2%, 0.4%, and 0.6% resulted in viscosity increases of 22.02%, 33.62%, and 33.55%, respectively. The A.0.4GO sample had the highest viscosity, slightly exceeding that of A.0.6GO. Variability across all aged samples was generally consistent, with both A.0.4GO and A.0.6GO showing similar mean values and variances.

**FIGURE 6:**
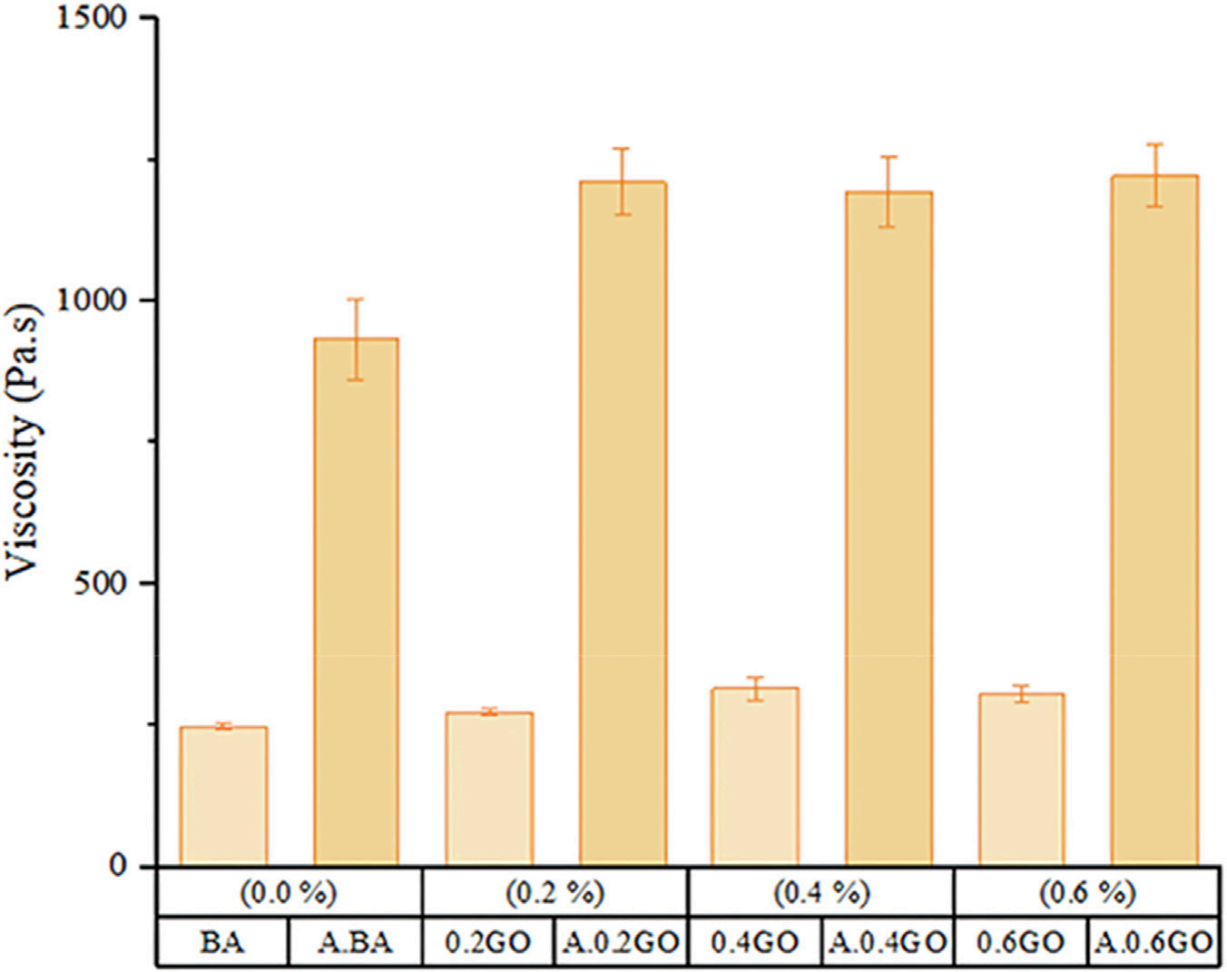
Viscosity comparison at 60 °C between aged and unaged asphalt samples.

Figure 6 compares the viscosity results of aged and unaged asphalt samples at 60 °C. Aging significantly increases the viscosity of all asphalt types. Specifically, the viscosity increase in aged samples compared to their unaged counterparts was 272.42% for A.BA, 310.96% for A.0.2GO, 322.04% for A.0.4GO, and 290.96% for A.0.6GO. These findings suggest that GO addition enhances the viscosity increase caused by aging, particularly at moderate concentrations.

The statistical analysis, using one-way ANOVA on the viscosity values at 60 °C in unaged asphalt samples, produced a p-value of 0.002. Since this is below the significance level of 0.05, the null hypothesis is rejected, confirming that there are statistically significant differences among the samples—specifically, BA, 0.2GO, 0.4GO, and 0.6GO. This finding demonstrates that adding GO has a considerable influence on the asphalt’s viscosity, highlighting the effect of this nanomaterial on a crucial performance parameter.

Further evaluation using Dunnett’s *post hoc* test showed that the 0.4GO (p = 0.008) and 0.6GO (p < 0.001) samples differed significantly from the BA. However, the 0.2GO sample did not show a statistically significant difference (p = 0.096). These results support a dose-dependent increase in viscosity with higher GO content, reaching a maximum at 0.6% GO. The analysis affirms that GO improves asphalt stiffness, with more noticeable effects at higher concentrations.

#### 3.3.2 Test 2: softening point

**FIGURE 7:**
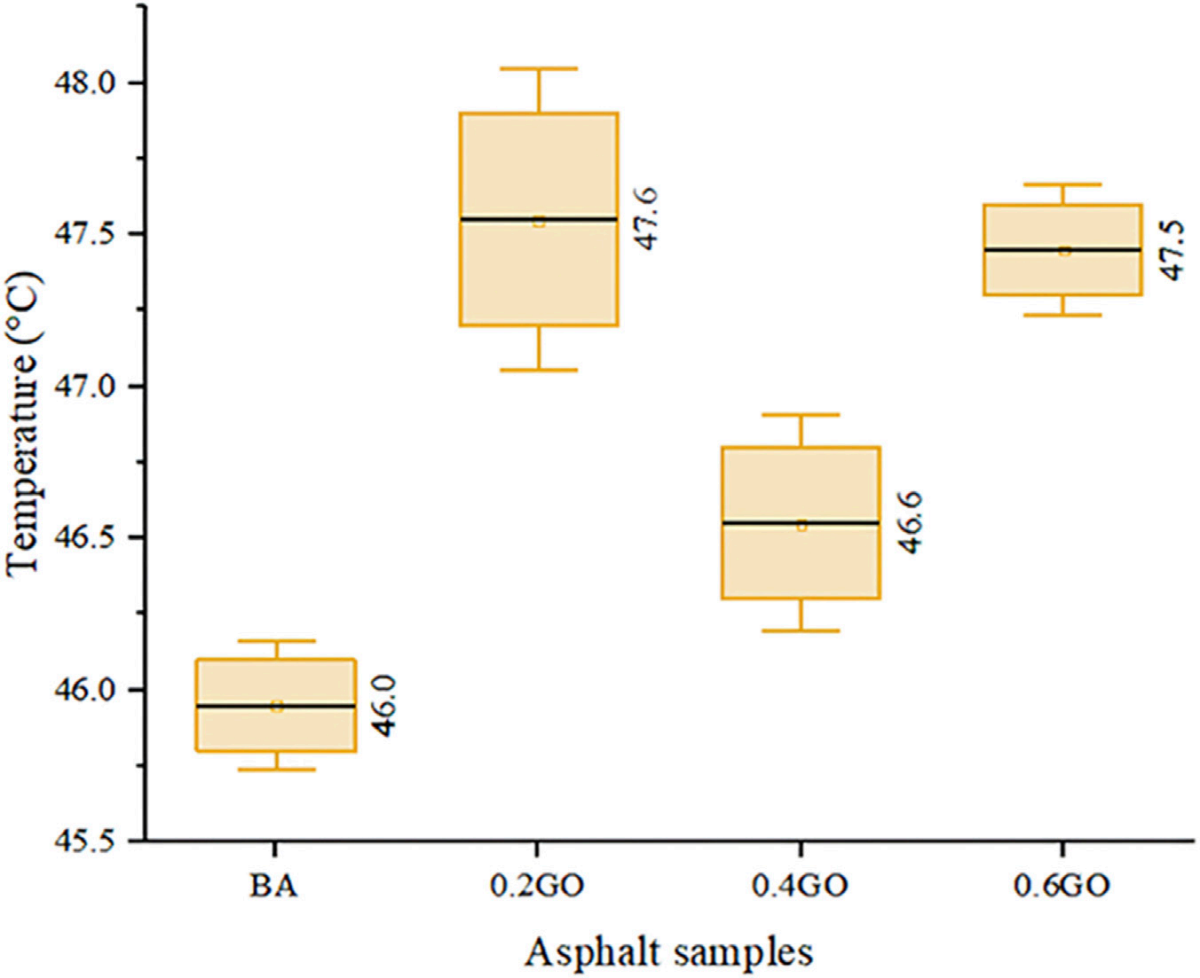
Softening point test results.

Figure 7 shows the softening point results for the asphalt samples. An irregular trend is observed, with softening point values generally increasing as GO concentration rises. This observation is consistent with previous findings in the literature (Wu et al., 2022; Zeng et al., 2017), although other studies have reported more linear trends in the increase of softening point with rising GO content (Jiang et al., 2022; Qian et al., 2022; Zeng et al., 2020; Zhang et al., 2022). When compared to the mean softening point of the BA sample, the incorporation of 0.2%, 0.4%, and 0.6% GO led to increases of 3.48%, 1.30%, and 3.26%, respectively. Interestingly, the highest softening point was recorded for the 0.2GO sample. It is important to note that this sample also exhibited considerable variance, which overlapped substantially with the results for the 0.6GO sample—an effect likely influenced by the inherent variability of the test method used.

The analysis of variance (ANOVA) performed on the softening point values yielded a p-value of 0.024, which is below the established significance threshold of 0.05. This result allows for rejecting the null hypothesis and confirms that statistically significant differences exist among the asphalt samples tested—BA, 0.2GO, 0.4GO, and 0.6GO. The findings demonstrate that the incorporation of GO has a measurable effect on asphalt’s resistance to softening at higher temperatures, highlighting the influence of GO on this important rheological property.

Post-hoc evaluation using Dunnett’s test showed statistically significant differences for most modified samples compared to the BA. However, the 0.4GO sample did not display a statistically significant difference (p = 0.308), indicating variability in the softening point response across GO concentrations. These findings highlight that although GO helps improve thermal stability, the effect is not linearly related to its concentration. Notably, the 0.2GO sample had the highest softening point among all variants.

#### 3.3.3 Test 3: penetration and penetration index

**FIGURE 8:**
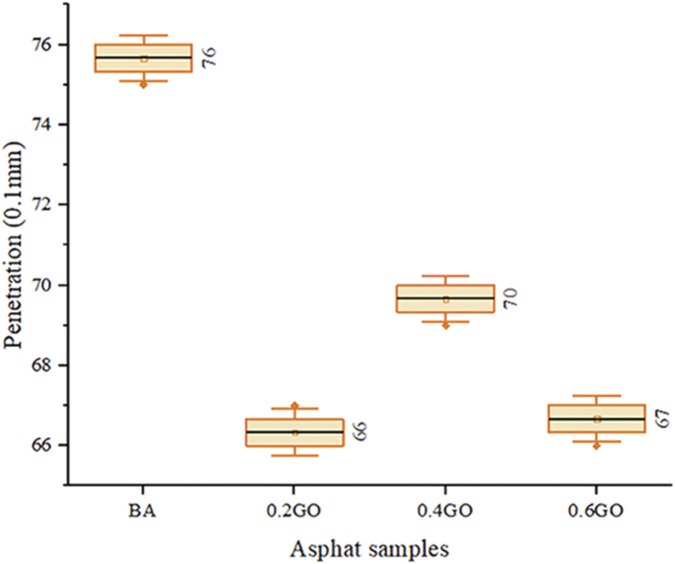
Penetration test results.

Figure 8 shows the results of the asphalt sample penetration tests, highlighting an overall decline in penetration values as GO content increases, although the trend is not perfectly linear. This finding is consistent with reports by Wu et al. (2022), while other studies have observed a more gradual and steady decrease in penetration with higher GO doses (Jiang et al., 2022; Qian et al., 2022; Zeng et al., 2017; Zhang et al., 2022). Compared to the average penetration of the BA sample, adding 0.2%, 0.4%, and 0.6% GO resulted in a decrease in penetration by 13.16%, 7.89%, and 11.84%, respectively. The 0.2GO sample had the lowest penetration, which was slightly less than that of the 0.6GO sample. Throughout all samples, variance remained relatively steady, indicating consistent test reproducibility despite performance differences.

The one-way ANOVA performed on the viscosity measurements at 60 °C showed a p-value of less than 0.001, which is significantly lower than the standard significance level of 0.05. This result supports rejecting the null hypothesis and confirms that there are statistically significant differences among the asphalt samples analyzed—namely BA, 0.2GO, 0.4GO, and 0.6GO. These findings clearly suggest that adding GO significantly affects asphalt hardness, highlighting GO’s influential role in this key performance property.

Subsequent Dunnett’s *post hoc* analysis showed that all GO-modified samples differed significantly from the base asphalt, with p-values less than 0.001 in each case. Interestingly, the increase in hardness was not linear across the GO concentrations, with the most significant improvement observed in the 0.2GO sample. This non-monotonic behavior indicates that while GO strengthens asphalt hardness, the relationship is not directly proportional to its amount.

#### 3.3.4 Test 4: ductility

**FIGURE 9:**
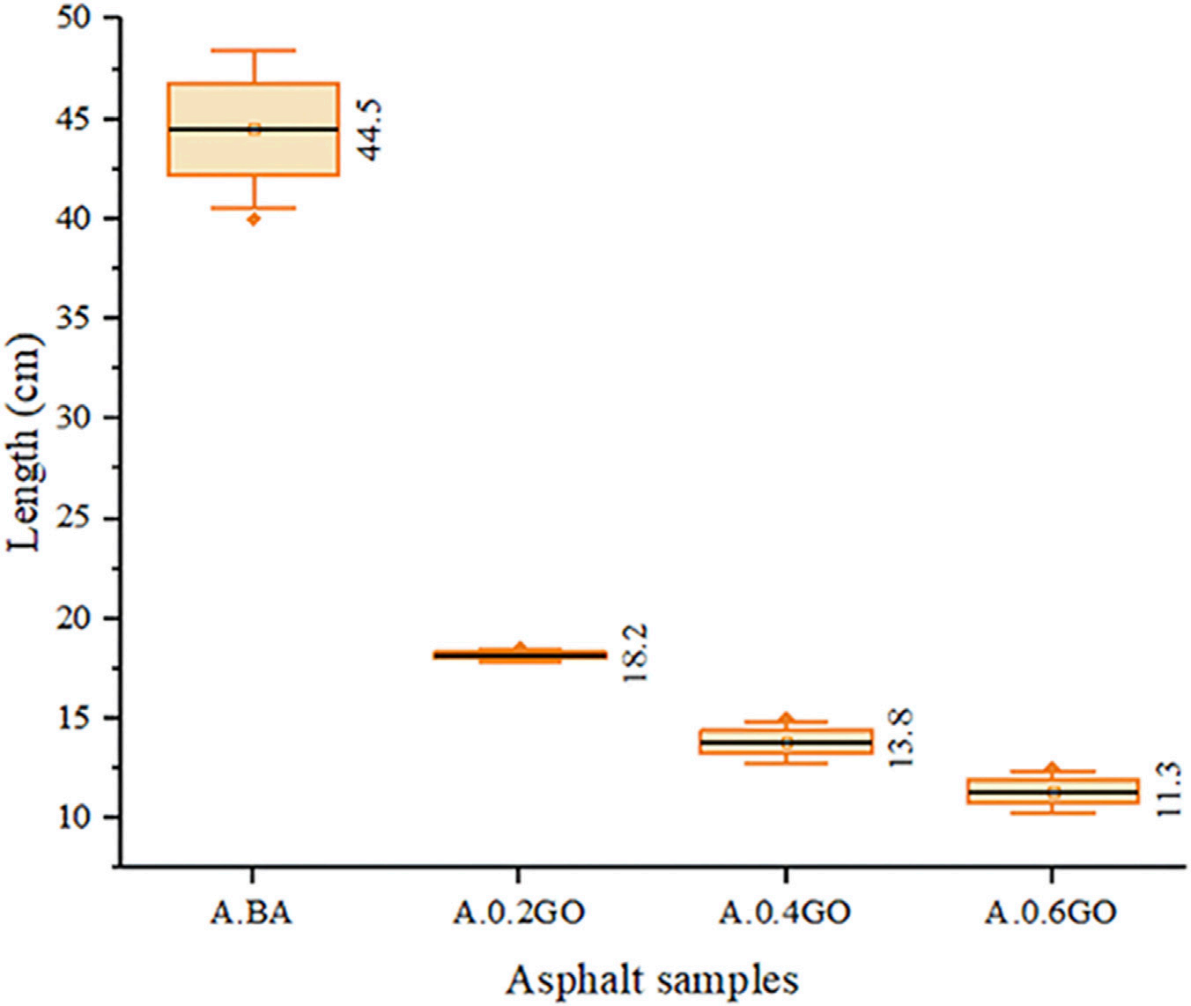
Ductility test results.

Figure 9 displays the ductility results of the aged asphalt samples. A marked and progressive reduction in ductility was observed as the GO content increased, consistent with findings reported in previous studies (Jiang et al., 2022; Zhang et al., 2022). However, this contrasts with the results of Qian et al., who documented a gradual improvement in ductility with increasing GO content (Qian et al., 2022). When compared to the mean ductility of the A.BA, the addition of 0.2%, 0.4%, and 0.6% GO resulted in reductions of 59.10%, 68.99%, and 74.60%, respectively. The lowest ductility was recorded for the A.0.6GO sample. Notably, the A.BA sample exhibited considerable variance, likely due to the sensitivity of the testing procedure, whereas the GO-modified samples demonstrated more consistent results across replicates.

The analysis of variance (ANOVA) conducted on the ductility values of aged asphalt samples showed a p-value of less than 0.001, which is significantly below the predefined significance level of 0.05. This allows for the rejection of the null hypothesis and confirms that there are statistically significant differences among the samples examined, specifically A.BA, A.0.2GO, A.0.4GO, and A.0.6GO. These results indicate that the addition of GO has a notable effect on asphalt ductility, demonstrating a direct impact of GO modification on this crucial mechanical property.

Dunnett’s *post hoc* analysis further confirmed these differences, as all GO-modified samples showed p-values below 0.001 when compared with the control (A.BA), indicating statistically significant reductions in ductility. The results clearly demonstrate a decreasing trend in ductility with increasing GO concentration, reaching the lowest value in the A.0.6GO sample. This supports the hypothesis that higher GO content can negatively affect the elasticity of asphalt, especially under low-temperature conditions.

#### 3.3.5 Test 5: specific gravity and density

**FIGURE 10:**
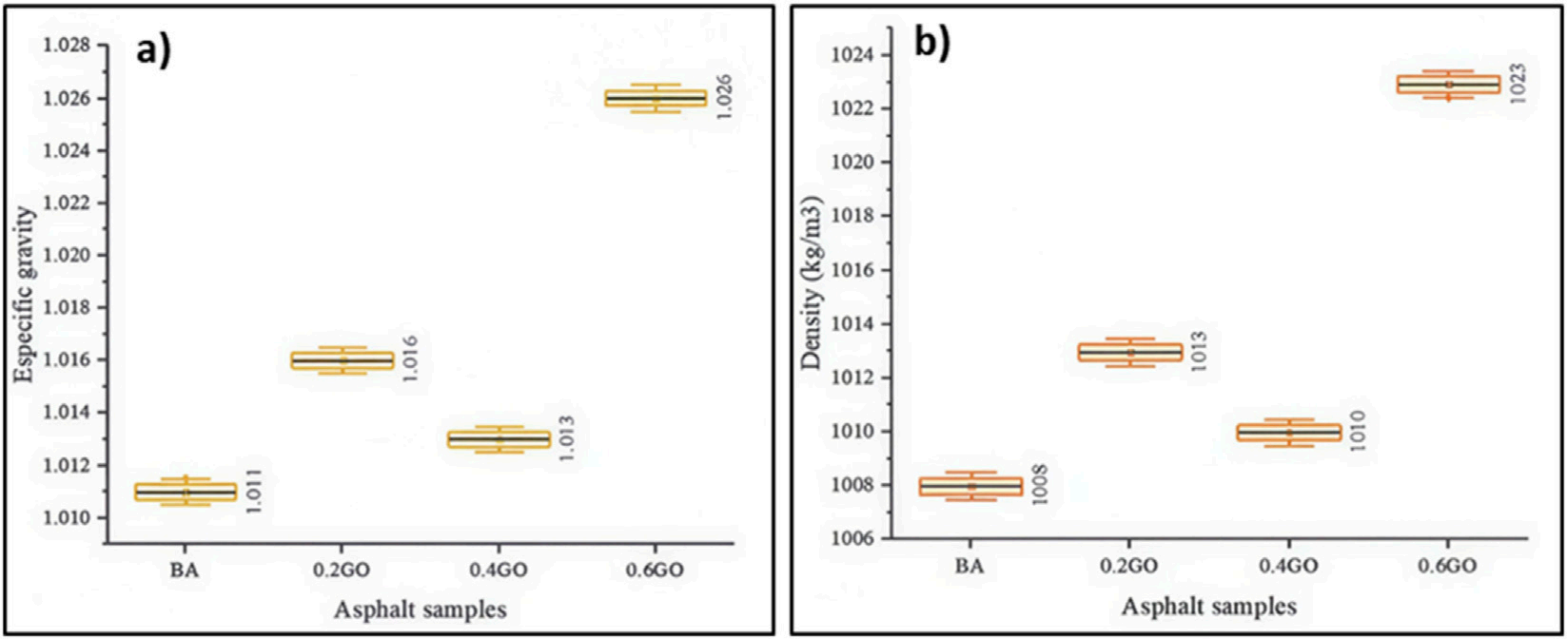
**(a)** Specific gravity and **(b)** density test results.

Figure 10a shows the specific gravity results for the asphalt samples. Because specific gravity is a dimensionless measure, no units are displayed on the axis label. The results reveal an irregular increase in specific gravity as GO content rises. Compared to the average value of the BA, adding 0.2%, 0.4%, and 0.6% GO increased specific gravity by 0.49%, 0.20%, and 1.48%, respectively. The highest specific gravity was observed in the 0.6GO sample. All samples showed similar variance, which aligns with the precision of the testing equipment used.

Figure 10b shows the density results, which reflect the trends seen in specific gravity due to the calculation method used. Density values increased irregularly with higher GO concentrations. Compared to the average density of the BA sample, adding 0.2%, 0.4%, and 0.6% GO resulted in increases of 0.50%, 0.20%, and 1.49%, respectively. Once again, the highest density was observed in the 0.6GO sample. Variance across all samples remained consistent, indicating reliable measurements under the testing conditions.

#### 3.3.6 Test 6: brookfield rotational viscosity at 135 °C

**FIGURE 11:**
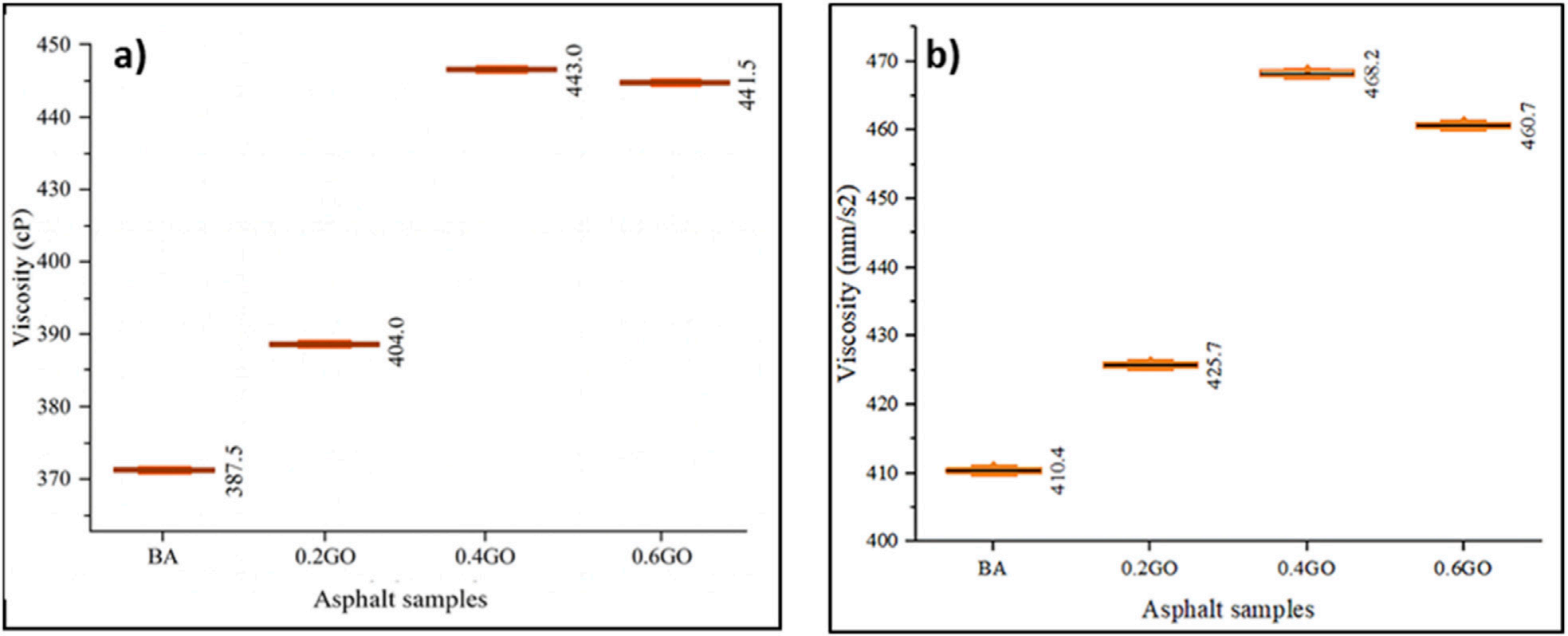
**(a)** Brookfield rotational viscosity and **(b)** kinematic viscosity test results at 135 °C.

Figure 11a shows the results of the Brookfield rotational viscosity test for the asphalt samples. An apparent increase in viscosity is observed with the gradual addition of GO, reaching a peak at the 0.4GO sample. Interestingly, viscosity decreases slightly when the GO content increases from 0.4% to 0.6%, a trend that aligns with findings reported in previous studies (Jiang et al., 2022; Wu et al., 2022). Compared to the mean viscosity of the BA, adding GO at 0.2%, 0.4%, and 0.6% resulted in increases of 4.26%, 14.32%, and 13.94%, respectively. The 0.4GO sample exhibited the highest viscosity, only slightly higher than the 0.6GO sample. Variance among all samples remained consistent, thanks to the precision and repeatability of the testing equipment.

Figure 11b shows the kinematic viscosity results at 135 °C for the same asphalt formulations. Similar to the rotational viscosity trend, kinematic viscosity increased with higher GO content, reaching a peak at 0.4GO before slightly decreasing at 0.6GO. Compared to the BA sample, kinematic viscosity increased by 3.73%, 14.08%, and 12.26% for the 0.2GO, 0.4GO, and 0.6GO samples, respectively. Again, the 0.4GO sample recorded the highest value, just above that of the 0.6GO sample. The variation across all samples was consistent.

#### 3.3.7 Test 7: flash and fire point

**FIGURE 12:**
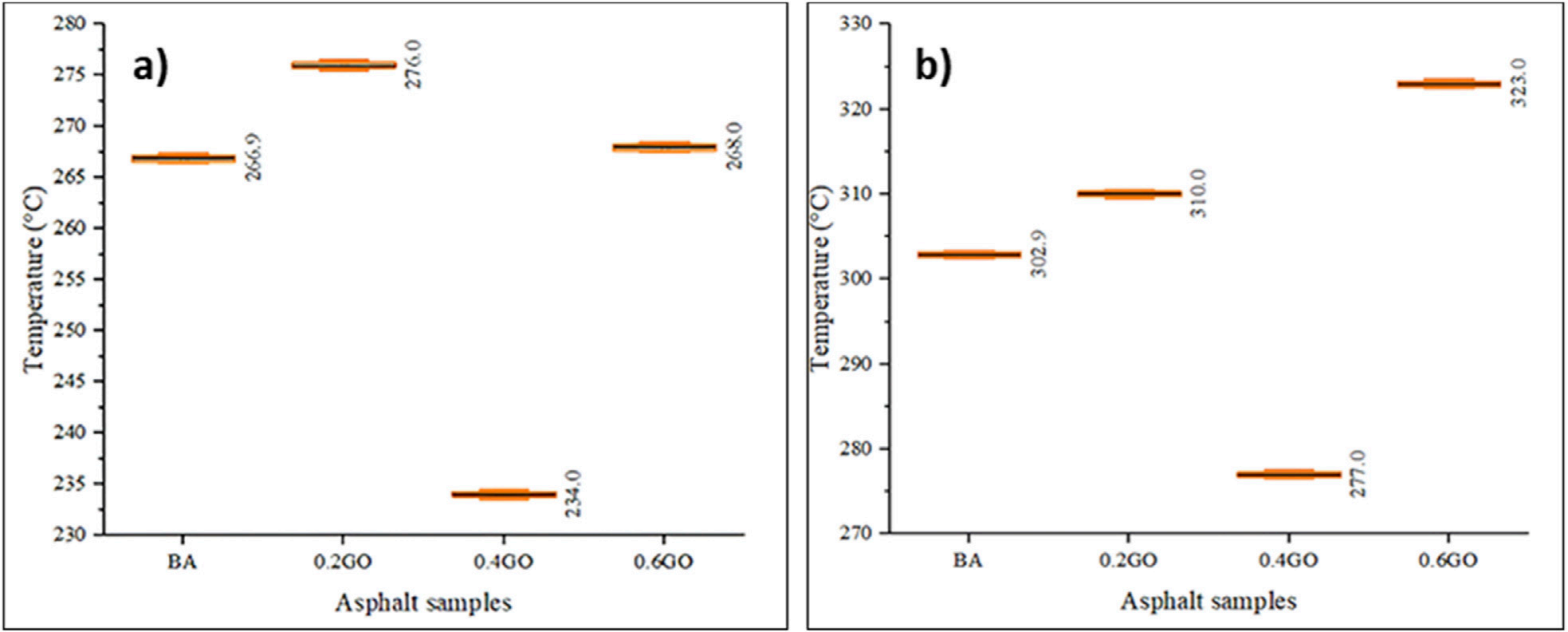
**(a)** Flash and **(b)** fire point test results.

Figure 12a shows the flash point results for the asphalt samples. Adding GO to the asphalt caused irregular changes in flash point values. Compared to the mean value of the BA, adding 0.2% and 0.6% GO increased the flash point by 3.41% and 0.41%, respectively. In contrast, the 0.4GO sample showed a significant decrease of 12.33%. Among all samples, 0.2GO had the highest flash point. The variation across all samples stayed consistent, thanks to the accuracy of the testing equipment.

Figure 12b shows the fire point results of the asphalt samples. Similar to the flash point results, fire point values also varied irregularly with increasing GO content. Relative to the BA sample, the inclusion of 0.2% and 0.6% GO increased the fire point by 2.34% and 6.64%, respectively, while the 0.4GO sample showed a decrease of 8.55%. Once again, the highest fire point was observed in the 0.2GO sample. Variability across all measurements was consistent, reflecting uniform test conditions and reliable instrumentation.

#### 3.3.8 Test 8: mass change

**FIGURE 13:**
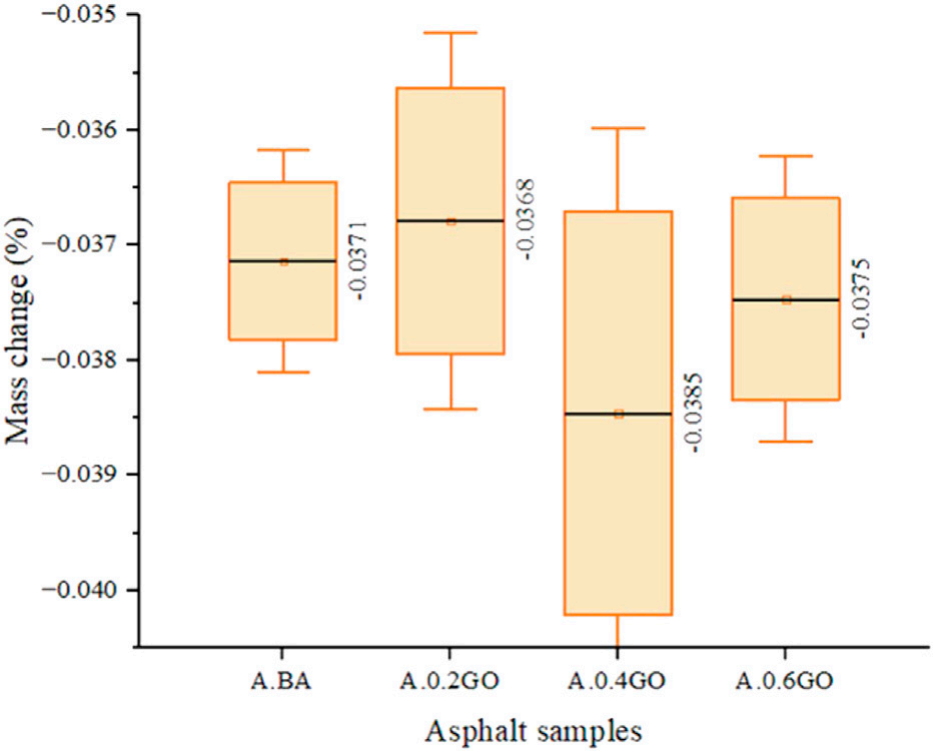
Mass change test results.

Figure 13 shows the mass change results for the aged asphalt samples. Adding GO caused irregular variations in mass change across different formulations. Compared to the average value of the A.BA, the addition of 0.4% and 0.6% GO increased the mass change by 3.77% and 1.08%, respectively. In contrast, the A.0.2GO sample showed a slight decrease of 0.81% in mass change. Among all samples, A.0.4GO had the highest value. Due to the testing method, all samples showed considerable variance, with overlapping value ranges across different GO concentrations.

## 4 Discussion

The integration of graphene oxide (GO) into AC-30 asphalt from the Esmeraldas Refinery showed different effects on various physical and rheological properties. Regarding viscosity, unaged samples exhibited a proportional increase with higher GO content, with the 0.6GO formulation achieving the highest absolute viscosity at 60 °C. Aged samples displayed a similar pattern across absolute, Brookfield rotational, and kinematic viscosities, but performance gains slowed after 0.4% GO, indicating a possible plateau for further improvements.

The softening point and penetration tests showed non-monotonic behavior. The highest softening point was observed for the 0.2GO formulation, while both 0.2GO and 0.6GO exhibited similar penetration values, indicating increased binder stiffness. Conversely, ductility decreased significantly as GO content increased, highlighting a decline in flexibility and raising concerns about performance at low temperatures.

The addition of GO also caused moderate increases in specific gravity and density, with peak values seen at 0.6GO. These results indicate a slight densification effect, which could be beneficial for compaction and long-term durability. However, flash and fire point values displayed irregular trends, especially for the 0.4GO sample, which deviated from expected performance. These irregularities, along with similar behaviors in mass change and other variables, highlight the complex interactions that may result from nanoparticle agglomeration, dispersion issues, or intermediate-phase chemical effects.

Despite such variability, consistent trends observed in the BA, 0.2GO, and 0.6GO samples confirm GO’s ability to improve stiffness, viscosity, and thermal resilience. These results align with established findings in composite material systems, underscoring the importance of precise control over additive dispersion and concentration when designing nanomodified asphalt binders.

To emphasize, within the scope of short-term aging (RTFO), GO influences both the initial condition and the apparent aging response. It increases pre-RTFO viscosity and does not reduce RTFO-induced hardening. This creates a trade-off between early rutting resistance (due to higher initial stiffness) and longer-term flexibility. A more comprehensive assessment of aging kinetics will require Pressure Aging Vessel (PAV) conditioning and Dynamic Shear Rheometer (DSR) parameters (e.g., MSCR and zero-shear viscosity) in future studies.

## 5 Perspectives and future work

For decision-making, modifiers should be benchmarked on a normalized basis—e.g., cost per unit increase in viscosity at 135 °C–165 °C, cost per unit change in multiple stress creep recovery (MSCR) non-recoverable creep compliance, or complex modulus—since unit prices and supply chains vary by region. At sub-percent loadings, GO is non-volatile and, due to our eco-friendly aqueous synthesis washed to near-neutral pH (≈6) and lacking solvent carriers, it is not expected to reduce binder flash or fire points; standard verification during scale-up (e.g., Cleveland Open Cup and Pensky–Martens flash point tests) will be included in pilot quality assurance/quality control (QA/QC).

Likewise, we do not expect additional fuming or odor issues from the modifier itself under normal mixing temperatures; plant trials will include routine emissions monitoring and odor observations along with throughput and compaction checks. No plant-mix trials were performed in this study; a staged pilot is planned to evaluate silo and mixer feeding, dispersion consistency, and any operational limitations. Powder-handling controls—such as enclosed feeding, local exhaust, and appropriate personal protective equipment (PPE)—will be used to reduce dust exposure during dosing. This framework places GO within an applied cost–performance range while maintaining the focus of the current study on early-stage thermal and rheological screening.

## 6 Conclusion

This study found that sub-percent graphene oxide (GO) modification of AC-30 under tropical conditions created a stiffer binder before aging and did not reduce RTFO-induced hardening. Overall, the results support immediate use as an early-rutting prevention measure in hot-climate wearing courses (e.g., intersections, bus lanes, steep grades), where higher initial stiffness is needed and construction temperatures must stay feasible for plant operations. The most practical operating range was 0.2–0.4 wt% GO, which provided measurable benefits without negative effects on flash/fire behavior. For implementation, the findings recommend routine QA/QC at 135 °C–165 °C (rotational viscosity), verification of softening point and penetration targets, and standard powder-handling controls during dosing. The work thus offers a quantitative foundation to start pilot plant trials of GO-modified AC-30 in tropical pavements, while also encouraging further evaluation of long-term aging (PAV/DSR) to balance early rutting resistance with maintaining flexibility.

## Data Availability

The raw data supporting the conclusions of this article will be made available by the authors, without undue reservation.
